# *In Vivo* Organic Bioelectronics for Neuromodulation

**DOI:** 10.1021/acs.chemrev.1c00390

**Published:** 2022-01-20

**Authors:** Magnus Berggren, Eric D. Głowacki, Daniel T. Simon, Eleni Stavrinidou, Klas Tybrandt

**Affiliations:** † Laboratory of Organic Electronics, Department of Science and Technology, Linköping University, 601 74 Norrköping, Sweden; ‡ Bioelectronics Materials and Devices, Central European Institute of Technology, Brno University of Technology, Purkyňova 656/123, 612 00 Brno, Czech Republic

## Abstract

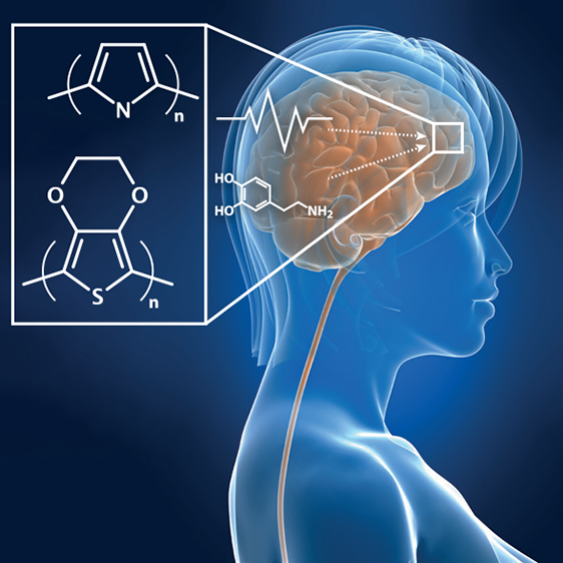

The nervous system poses a grand challenge for integration with modern electronics and the subsequent advances in neurobiology, neuroprosthetics, and therapy which would become possible upon such integration. Due to its extreme complexity, multifaceted signaling pathways, and ∼1 kHz operating frequency, modern complementary metal oxide semiconductor (CMOS) based electronics appear to be the only technology platform at hand for such integration. However, conventional CMOS-based electronics rely exclusively on electronic signaling and therefore require an additional technology platform to translate electronic signals into the language of neurobiology. Organic electronics are just such a technology platform, capable of converting electronic addressing into a variety of signals matching the endogenous signaling of the nervous system while simultaneously possessing favorable material similarities with nervous tissue. In this review, we introduce a variety of organic material platforms and signaling modalities specifically designed for this role as “translator”, focusing especially on recent implementation in *in vivo* neuromodulation. We hope that this review serves both as an informational resource and as an encouragement and challenge to the field.

## 1 Introduction

The nervous system consists of various components that process and transfer signals, which in turn regulate and actuate internal functions as well as record internal and external sensory information.^1^ In vertebrates, the peripheral nervous system (PNS) consists of nerve bundles that are composed of many axon fibers that transport outbound and inbound signals, at speeds around 100 m/s, to and from the central nervous system (CNS). In the CNS of a human approximately 10^11^ somas (neuron cell bodies) together define the central signal processing unit. In the CNS, every soma captures signals via its dendrite branches and releases its signals through its axon cable, ending with the telodendrion. At the boundaries between the telodendrions and “downstream” somas, in total more than 10^14^ synapses process and transmit neural signals. This signaling of both the CNS and PNS includes a complex combination of electric, ionic, chemical, and structural features.

Precise regulation of neuronal function, in both the PNS and CNS, is a grand challenge and is highly anticipated as many long-standing questions of neurobiology remain unanswered due to a lack of proper signal triggering technology. For example, deep brain stimulation has been available for over 25 years and is widely used in the clinic,^2^ but the underlying mechanisms of action remain unclear and the devices themselves have seen only marginal technological advances since their introduction.^3,4^ In addition, several proposed neural prosthetic and therapeutic techniques are hampered since adequate stimulation, electrode resolution, and multifunctional interaction with neuronal signaling are still not possible.^5,6^ To enable such manipulation and control of the signaling cascades of the PNS and CNS, a technology with proper addressing, complexity, speed, and miniaturization is needed that can “speak the language” of depolarization and neurotransmitters. Neuromodulation traditionally relies on the injection of electrolytic charge from a solid-state electrode. The concept of electrical to ionic transduction is at the center of any bioelectronic interface.

Of all human-made technologies with signaling characteristics that can match those of the CNS and PNS, complementary metal oxide semiconductor (CMOS) based electronics and solid-state photonics are the only ones readily at hand.^7^ However, there is a fundamental challenge in connecting analog or digital solid-state Si-based circuitry directly^8^ to the nervous systems due to a lack of signal translators which can convert an electronic addressing signal into the expression of signal entities that can be received and interpreted by the components of the CNS and PNS, i.e., the synapses, nerve bundles, etc.

**Figure 1:**
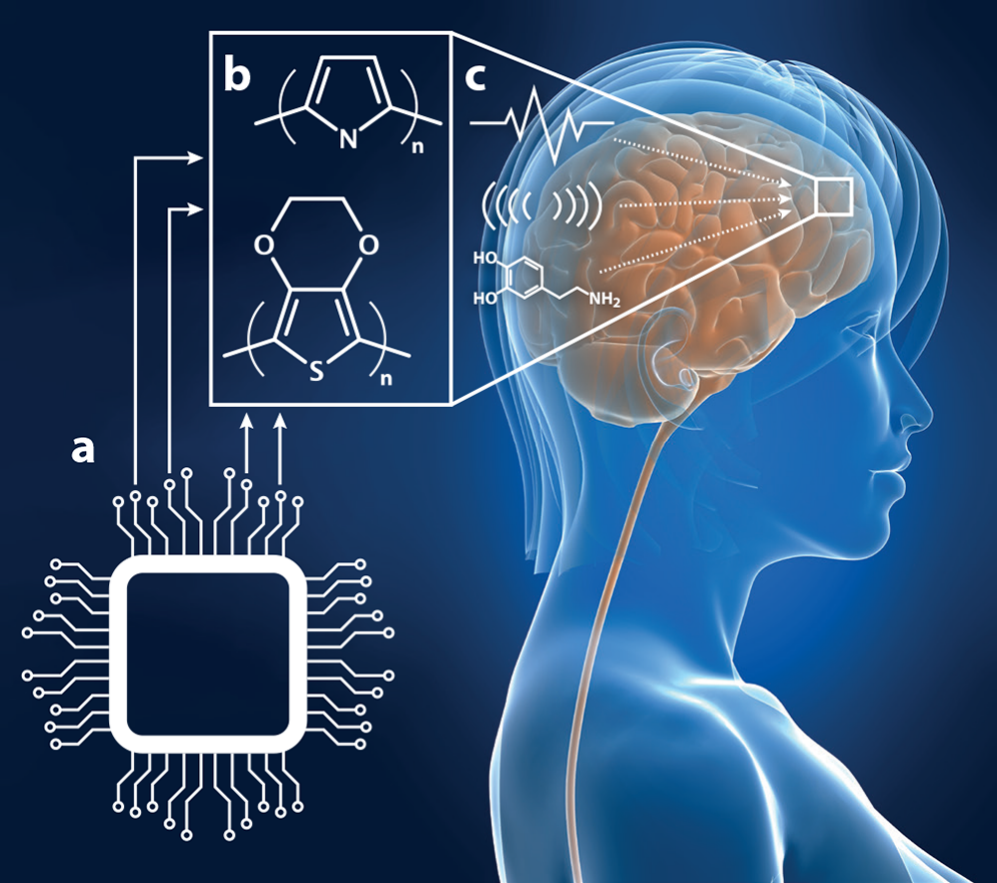
*In vivo* organic bioelectronic neuromodulation. (a) Multiple, parallel electronic signals can be transduced using (b) organic electronic materials, electrodes, and structures. (c) The broad array of neuromodulatory signals arising from the organic electronics can include, e.g., electrical, physical/piezoelectric, or biochemical stimuli.

Organic electronic materials and devices represent a key enabling technology that possesses many of the desired features for translating electronic signals into the endogenous signaling entities of the PNS and CNS (Figure 1). In this paper, we review the early and recent progress on the topic of developing neurostimulation devices based on organic materials, specifically targeting *in vivo* applications. We focused on reviewing organic bioelectronics^9^ in the form of electrodes, devices, and systems, with specific features related to elasticity, signal translation fundamentals, proximity, biostability, biocompatibility, self-organization, and more, in an attempt to make the technology–nervous system signaling interface seamless. Organic bioelectronics are defined as those based on organic semiconducting and conducting materials comprising conjugated organic molecules. This includes molecular materials such as macrocycles, up to and including conjugated polymers. This definition excludes materials based on allotropes of carbon such as nanotubes, graphene, diamond-like carbon, etc. We hope this review serves both as a source of information and as a benchmark and encouragement for further developments.

## 2 Coatings for Existing Electrodes

Polymers were first identified in the early 1980s^10^ as a key material in neurostimulation applications—before the era of organic electronics—owing to their flexible, permeable,^11^ biocompatible, and inert characteristics.^12^ For instance, platinum-on-tantalum electrode arrays were photolithographically defined and sandwiched between thin polyimide layers,^12^ with access openings produced for electrode stimulation. The resulting Kapton device was inserted through the round window of the inner ear, and successful cochlear prosthesis operation was demonstrated. However, the rise of intrinsically conducting polymers,^13,14^ and stable characteristics while operating in aqueous media, opened up radically new opportunities of defining electrodes combining several anticipated “plastic” properties with electroactivity and amalgamating desired mechanical and biochemical features with electronic characteristics and functionalities. Suggestions for using conjugated polymer electrodes (CPEs) to record or regulate functions of neurons was suggested and presented as early as 1991.^15^ In a few early studies, neuronal cells were applied to conjugated polymer coatings or electrodes in an attempt to explore biocompatibility and regeneration of nervous tissue,^16^ ultraflexible neural intrafascicular electrodes,^17^ and neurite outgrowth.^18^ In particular, polypyrrole (PPy) was examined in an *in vivo* experiment in 1994.^16^ Here, various forms of PPy electrodes were examined, such as PPy added directly onto Pt wires and then implanted into a rat model with a minimal tissue response observed 4 weeks after surgery. Further, PPy-based CPEs were also examined to trigger and regulate angiogenesis (regeneration of blood vessels) *in vivo*.^19^

**Figure 2:**
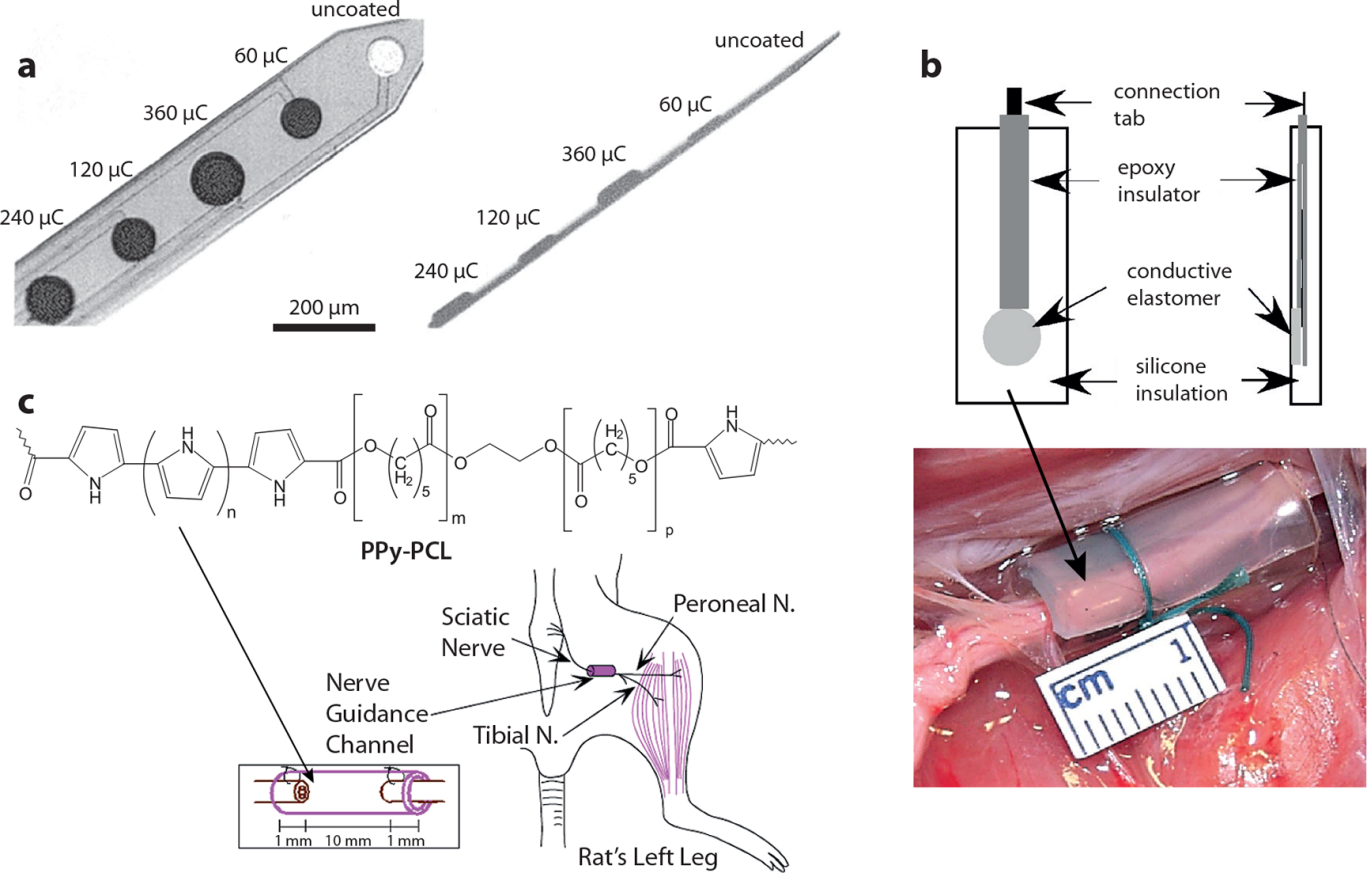
(a) Surface-modified depth probes with improved recording capability. The charges indicate the amount of electropolymerization of PPy:PSS. (b) Nerve-cuff electrode featuring nanoparticulate PPy within silicone elastomers. (c) PPy–PCL copolymer degradable tubular electrode for regeneration of the sciatic nerve. Part a reproduced with permission from ref (20). Copyright 2001 John Wiley and Sons. Parts b and c reproduced with permission from refs (31 and 32), respectively. Copyright 2007 and 2010 Taylor & Francis.

The achievements listed above blazed the trail for the work to derive dedicated CPEs applied *in vivo* to record and regulate neuronal signaling and tissue (re)growth/generation. A first step was taken in 2001, when Martin and co-workers reported surface-modified neural electrodes with improved recording capability.^20,21^ Micromachined silicon probes with gold electrodes were coated with PPy combined with polystyrenesulfonate (PSS) or biomolecules from aqueous solutions. The PPy phase was galvanostatically grown at a current density of 0.5 mA/cm^2^, reaching a total passed charge ranging from 60 to 240 μC (see Figure 2a). The resulting “fuzzy” electrode morphology, provided by the PPy cladding, exhibited a more efficient interface for electronic and ionic signal transport, and the biomolecule coating with cell-binding functionality also offered improved cell attachment. Soon after this achievement, the Inganäs team reported conducting hydrogel CPEs based on PEDOT:PSS (poly(3,4-ethylenedioxythiophene) doped with PSS) manufactured onto a micromachined polydimethylsiloxane (PDMS) substrate.^22^ In this work, the emphasis was aimed to develop an all-flexible device expressing a high capacitance value, per area active electrode, optimized for signal recordings, along with elastic properties similar to those of the targeted tissue or brain. Several early studies also aimed to investigate the overall biocompatibility,^23^ biostability, and interaction with proteins under electrical stimulation from CPEs *in vivo*.

The work on CPEs to regulate and record cell functions and neuronal signaling was thus established, and several groups subsequently entered this research effort.^24^ In 2004, the first steps toward using conjugated polymer electrodes for *in vivo* neurostimulation of the CNS and PNS was reported.^25^ At the same time, it was also shown that electropolymerized PPy on nylon/spandex fabric electrodes exhibited successful *in vivo* electrotherapeutic results when they were applied to a neuropathic pain animal model.^26^ Soon after, refined and dedicated CPEs were frequently developed and explored, such as for regulating nerve regeneration on biodegradable composites (PPy doped with butanesulfonic acid)^27^ and improving the nerve–electrode interface of cochlear implants (PPy doped with *p*-toluenesulfonate coated on Au).^28^ Further work investigated the necessary biostability and biocompatibility of conducting polymers, such as by investigating the short-term histocompatibility and signal throughput^29^ and by incorporating polysaccharides (heparin) as dopants to limit PEDOT’s immunological response in cortical tissue.^30^ In an attempt to derive CPEs with tailor-made elastic properties, nanoparticulate PPy was polymerized within silicone elastomers and then shaped into a cuff-electrode configuration (Figure 2b).^31^

CPE materials can be manufactured and shaped into mechanical, structural, and functional systems, which provide great freedom to define dedicated electrode settings for specific *in vivo* neurostimulation applications. PPy–poly(ε-caprolactone) (PCL) copolymers were for instance synthesized and explored as degradable electrodes for regeneration of the sciatic nerve (Figure 2c). After 8 weeks from implantation, the tubular electrode including the biodegradable PPy–PCL cladding contained a healthy nerve cable and no inflammatory response was observed.^32^ PEDOT-coated PtIr and IrOx electrodes were also found to have superior signal-to-noise recording and charge injection characteristics when they were evaluated by using electrochemical impedance spectroscopy, both *in vitro* and when implanted in rat cortex.^33^ A similar study was conducted for PEDOT coated on bare Pt microelectrodes, which confirmed previous reports.^34^ In an attempt to further “open up” the electrode structure, vapor phase polymerization of PEDOT was applied to a 3D microparticle assembly. The microparticles were then selectively excluded, which rendered the resulting electrode highly porous with voids defined on the micrometer scale.^35^ In an attempt to derive electrodes that mimic the structure and morphology of the targeted neuronal system, to reach a seamless electro-neuro interface, *in situ*/*in vivo* polymerization of PEDOT using iron chloride was conducted.^36^ The resulting electrode was produced inside acellularized muscle tissue constructs, and a resulting tissue–electrode amalgamation was thus achieved. A similar approach was later used to electropolymerize EDOT monomers to form a PEDOT cloud electrode with a protrusion penetrating brain tissue^37^ and the hippocampus of live rats.^38^

Recent work on the development of CPEs for *in vivo* neurostimulation has been devoted to deriving highly sophisticated electrode devices and systems. For instance, PEDOT doped with PSS-*co*-(maleic acid) (PSS-*co*-MA) was coated on carbon microfibers (7 μm in diameter) forming an intraspinal microstimulation (ISMS) scaffold. The ISMS electrode was introduced into the cervical spinal cord of anesthetized rats, and successful activation of specific spinal motor neurons was achieved, with an increased activation response for PEDOT:PSS-*co*-MA coated carbon fibers compared to noncoated ones.^39^ New material formulations have also been explored recently, such as PEDOT:Nafion with an improved charge injection limit reaching 4.4 mC/cm^2^.^40^

## 3 Chemical Stimulation and Drug Delivery

### 3.1 Controlled Delivery Electrodes

**Figure 3:**
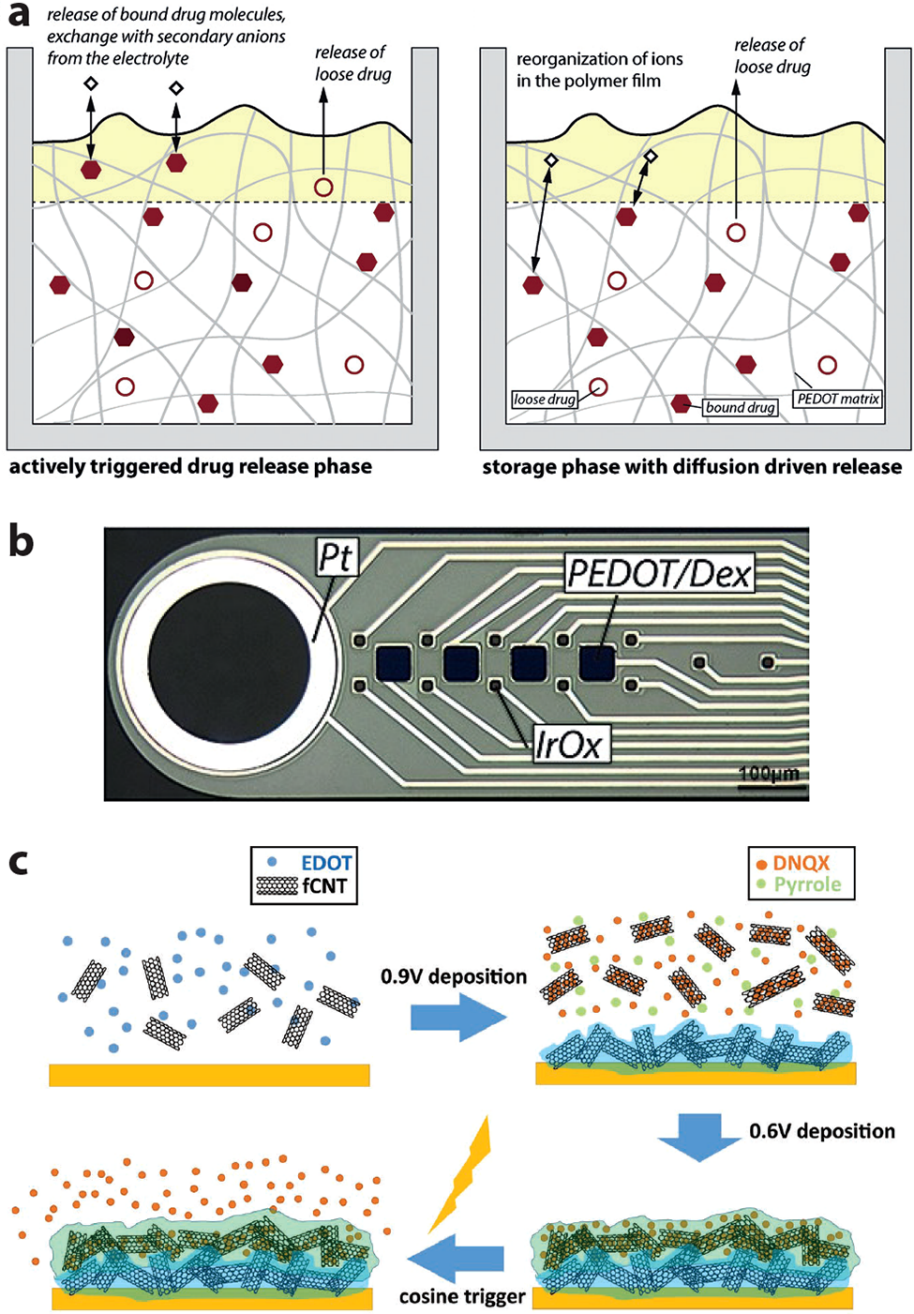
Controlled release via conducting polymer electrodes. (a) Canonical mechanism with active (left) and passive/diffusion (right) drug release. (b) Combination of controlled release (anti-inflammatory dexamethasone, Dex) and neural recording (large electrode to left) on a single flexible depth probe. (c) Enhancement of controlled release using functionalized carbon nanotubes. Parts a and b reproduced with permission from refs (50 and 54), respectively. Copyright 2019 and 2017 Elsevier. Part c reproduced with permission from ref (55). Copyright 2018 John Wiley and Sons.

The mixed ionic and electronic properties of organic electronic materials make them a promising platform of controlled substance release technologies. In the most straightforward embodiment of controlled drug release from organic electrodes, the ions associated with doping and charge compensation along the polymer backbone can be released from the electrode during electrochemical switching. For example, in the electrochemical switching of the archetypal PPy from its oxidized (charged) to neutral state, the compensating counterion (A^–^) is released into bulk solution: PPy^+^:A^–^ + e^–^ → PPy^0^ + A^–^. Indeed, chemical delivery has been an integral part of the field of conducting polymers since the early 1980s. As early as 1984, Zinger and Miller were investigating the release of the neurotransmitter glutamate from PPy films.^41^ The appeal of such controlled release is obvious: if the bioactive compound can be contained by the organic electrode and released on demand by simple voltage pulses, a precise and local drug delivery system can be achieved and provide an alternative to problematic systemic dosage (injections, pills, and the associated side effects) or fluidic delivery (requiring complicated pumps and plumbing). Such controlled delivery has been particularly appealing in the realm of neuroscience, since the target cells and tissue are particularly sensitive to chemical and physical (e.g., fluidic pressure) changes in their environment. Over the decades, a variety of such controlled delivery electrodes have been demonstrated, all following the basic recipe of embedding bioactive substances in the redox-active conducting polymer film (as counterions in the PPy example above, or co-ions compensating the counterions) and releasing them into the adjacent electrolyte upon redox switching (Figure 3a). These devices have been extensively reviewed elsewhere^42−45^ and have been utilized in a variety of recent *in vitro* neuromodulation demonstrations.^46−52^ However, such controlled delivery electrodes have seen few neuromodulation demonstrations *in vivo*.

In 2016, Wallace *et al.* demonstrated a “pre *in vivo*” system combining electrocorticography (ECoG) signals associated with epilepsy as the input signal for controlled release of the antiepileptic drug fosphenytoin (FOS).^53^ They used prerecorded human ECoG signals to trigger release of FOS from a PPy film on a quartz crystal microbalance (QCM) to validate the “seizure initiated” system. Using a constant-current delivery protocol to ensure consistency, they demonstrated a latency (between exceeding the ECoG threshold and actual drug release) of only ∼10 s. In 2017, Asplund *et al.* combined classic conducting polymer controlled delivery with flexible neural depth probes.^54^ In that study, they embedded the anti-inflammatory dexamethasone (Dex) in multiple PEDOT electrodes on the same flexible polyimide-based recording probes (Figure 3b). The aim was to mitigate the inflammatory response common to such depth probes which, over time, causes degradation of recording quality. Over the course of 12 weeks, they were able to periodically release Dex precisely at the implant location and observe that active neurons did indeed remain closer to the recording electrode. While the neuron–electrode proximity was only marginally affected, the study did prove the concept of relatively long-term implantation of conducting polymer-controlled delivery electrodes. Meanwhile, Cui *et al.* have been experimenting with nanostructured additives to enhance delivery performance *in vivo*. In 2018, they used functionalized carbon nanotubes in a combined PEDOT and PPy film to deliver 6,7-dinitroquinoxaline-2,3-dione (DNQX), a competitive agonist to glutamate receptors, into the barrel cortex of rats (Figure 3c).^55^ Using multielectrode depth probes modified with their delivery electrode material, they were able to demonstrate the expected neural suppressive effect of DNQX up to 446 μm from the release site. More recently, the team has demonstrated a PEDOT-based delivery electrode incorporating mesoporous sulfonated nanoparticles for enhanced drug loading.^56^ With these devices, they increased the charge injection limit (for electrical stimulation), increased the drug loading capacity by 16.8 times compared to pure PEDOT, and again demonstrated *in vivo* neural suppression via DNQX delivery (in mouse brain).

### 3.2 Iontronic Delivery

**Figure 4:**
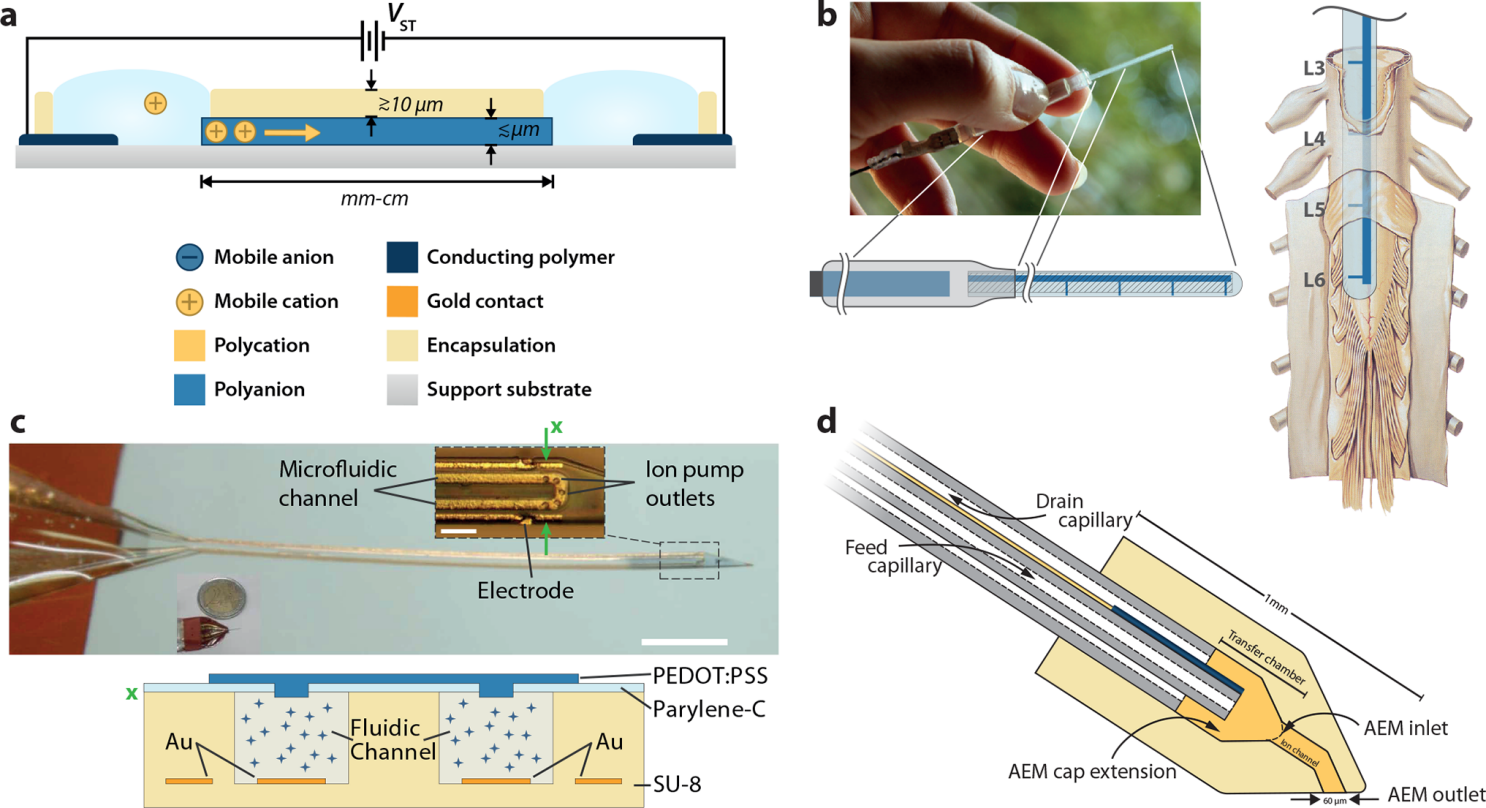
Iontronic drug delivery. (a) Basic lateral organic electronic ion pump (OEIP) with characteristic length scales. In this depiction, the source (anodic) electrolyte is on the left and the target (cathodic) electrolyte is on the right. (b) OEIP adapted for delivery of GABA directly to the relevant nerve junctions on the rodent spinal cord, for pain therapy. (c) Microfluidic ion pump (μFIP) adapted for depth probe implantation. The scale bar is 1 mm, and the cross section at “×” is depicted below. (d) Freestanding fluidic capillary-based device with “iontronic cap”. Parts a and d reproduced with permission from refs (57 and 74), respectively. Copyright 2018 and 2021 John Wiley and Sons. Parts b and c reproduced with permission from refs^71 and 72^, respectively. Copyright 2015 and 2018 AAAS.

Another version of drug delivery utilizing the unique ionic properties of organic electronics has emerged in the 2000s in the form of so-called “iontronics”.^57,58^ Iontronics represent circuits, circuit branches, and components where the dominant or exclusive charge carriers are ions rather than electrons or holes. The concept is an extension of ion exchange systems whereby ions are transported through selective membranes by the application of electric fields (electrophoresis). Iontronics, in this context, are typified by their original demonstration in 2007: the organic electronic ion pump (OEIP),^59^ effectively an iontronic resistor (i.e., ionic current directly proportional to applied voltage). This original OEIP was based on a single thin film of the well-known PEDOT:PSS with regions of the PEDOT component “deactivated” by chemical overoxidation, leaving ionically conducting (but electronically insulating) regions of polyanionic PSS. In the fully hydrated state, cations could be “pumped” electrophoretically laterally across the PSS region, from a “source” electrolyte to a “target” electrolyte (Figure 4a). The polyanionic nature of the PSS rendered it a lateral (several millimeters) cation exchange membrane (CEM), blocking the electrophoretic flow of anions from the target toward the source. In this way, the OEIP represents a platform for charge-selective delivery of small- to medium-sized ionic compounds on demand (no delivery in the absence of applied voltage) and without liquid flow (aside from hydration sheaths, no liquid is “pumped” along with the ions). As with the controlled delivery electrodes above, such spatiotemporally resolved delivery, without liquid flow, is of great appeal in the realm of neuromodulation. From the first OEIP demonstrations,^59,60^ delivering neuroactive compounds for neuromodulation purposes has been a top priority. Over the years, a variety of devices have been demonstrated using CEMs for cationic drug delivery and anion exchange membranes (AEMs) for anionic drug delivery, in a variety of form factors and by various research groups.^43,57,61,62^ Additional iontronic components and circuits have also been developed, such as diodes,^63^ capacitors,^64^ transistors,^65−67^ rectifiers,^68^ and logic circuits.^69^

In 2009, Simon *et al.* demonstrated the first *in vivo* application of OEIPs, for modulating auditory function in a guinea pig model.^70^ In these experiments, the planar geometry of previous OEIPs was encapsulated by using medical-grade tubing (over the source and target electrolytes) and PDMS over the tapered “delivery tip”. Devices were mounted on the round window membrane of anesthetized guinea pigs, and auditory function was modulated by delivery of glutamate which elicited a selective excitotoxic effect on the inner hair cells of the cochlea. This acute excitotoxic demonstration paved the way for a follow-up project focusing on treating neuropathic pain. In 2015, a simplified OEIP implant specifically designed to match the physiology of the rat spinal cord was demonstrated.^71^ In these experiments, the OEIP was designed with four outlets connected as a parallel iontronic circuit and arranged to match the position of the L3–L6 dorsal roots, where the sciatic nerve bundles enter the spinal cord and relay the pain signal into the CNS (Figure 4b). Delivery of the inhibitory neurotransmitter γ-aminobutyric acid (GABA) at these specific points (to awake animals) resulted in a significant reduction of the pain threshold (von Frey filament test in a spared sciatic nerve model) while only requiring approximately 1% of the required dosage used in intrathecal injection. Around this time, Malliaras and Proctor *et al.* began development of a hybrid microfluidic ion pump (μFIP) architecture combining the long-range versatile delivery of fluidics with the high-resolution spatiotemporal delivery of OEIPs. In 2017, they demonstrated the first μFIP, based on a conformable parylene C based microfluidic architecture with vertical CEM outlets (based on PEDOT:PSS) in the “roof” of the fluidic channel.^61^ The devices exhibited ideal iontronic performance—low leakage and low voltage operation—and were successfully used to deliver K^+^ to the cortex of anesthetized rats, eliciting the expected hyperexcitability (increased spiking). In 2018, they demonstrated a μFIP adapted for implant application (depth probe, Figure 4c).^72^ This device was used to deliver GABA into the hippocampus of anesthetized mice in a 4-AP epilepsy model. GABA delivery via the μFIP successfully suppressed pathological neural spiking events (only when actively delivering, not during passive delivery), demonstrating for the first time “deep” *in vivo* neuromodulation using ion pump technology. Most recently, Proctor *et al.* have demonstrated an integrated sensing and drug delivery platform using the conformable parylene C based μFIP combined with a PEDOT:PSS-based ECoG electrode array surrounding the ion pump outlets.^73^ Using an anesthetized mouse cortex model, they were able to successfully demonstrate the *in vivo* delivery of various neuro-active compounds with simultaneous—and importantly, colocalized—electrophysiological recording. The hybrid microfluidic ion pump concept has also been demonstrated by using a free-standing capillary fiber form factor. In 2021, Arbring Sjöström *et al.* demonstrated a coaxial capillary device (for fluid inlet and outlet) with an “iontronic cap” featuring an AEM ion channel (Figure 4d).^74^ With the PEDOT-based source electrode (cathode) incorporated onto the surface of the inner capillary, a minimal amount of wiring was needed to drive the device (the anodic counter electrode was a separate piece of PEDOT:PSS), making it simple to mount on a micromanipulator and integrate into a standard electrophysiology setup. Glutamate was delivered (as an anion) into artificial cerebrospinal fluid and used to demonstrate the spatiotemporal precision (via a glutamate biosensor), paving the way for application in brain slice models or as a depth probe.

In addition to the basic OEIP functionality described above, i.e., operation as an iontronic resistor, the analogy between the majority cationic and anionic carriers in CEMs and AEMs and the holes and electrons in p- and n-type semiconductors has enabled the development of a range of iontronic components including bipolar membrane diodes (CEM–AEM junction)^63^ and bipolar junction transistors (AEM–CEM–AEM stack),^65^ as well as analog^64^ and digital^63,66^ ionic circuits. While these more complex devices have yet to reach *in vivo* applications, they have enabled significantly advanced functionalities that pave the way for more advanced neuromodulation. For example, the integration of vertical iontronic diodes along the length of lateral OEIP channels enabled individually addressable neurotransmitter delivery with on/off switching of ∼50 ms (as opposed to ≳1 s for lateral or fluidic-based OEIPs).^75^ More recently, purely geometric modifications of the ion channel encapsulation have enabled “polarization diodes” to reach on/off switching of ∼1 ms, finally approaching the temporal dynamics of synaptic transmission.^76^ Finally, integration of palladium-based “proton traps” along the length of lateral ion channels has enabled a significant increase in delivery efficiency (ratio of delivered neuroactive compound to electronic charge in driving circuit, with the ideal case of 1:1).^77^

## 4 Photonic Approaches

Organic semiconducting materials can be highly efficient light absorbers.^78^ Organic semiconductors have, relative to their inorganic cousins, remarkably high optical absorption coefficients. Poly(3-hexylthiophene), P3HT, is an archetypical polymeric p-type semiconductor used extensively in organic photovoltaics. In the region of strongest absorption between 400 and 600 nm, P3HT has an absorption coefficient of between 1 × 10^5^ and 5 × 10^5^ cm^–1^. Phthalocyanines, which are macrocyclic small molecules deployed in vacuum-processed organic photovoltaics, have absorption coefficients of the same magnitude. Metal-free phthalocyanine, H_2_Pc, has a peak absorption coefficient of 3 × 10^5^ cm^–1^ at 650 nm.^79^ Silicon, the standard inorganic photovoltaic material, has an absorption coefficient of 3 × 10^3^ cm^–1^ at 650 nm, a 100-fold difference. Absorption coefficients of organic semiconductors such as P3HT and H_2_Pc even exceed those of highly efficient absorbing direct-band-gap materials such as germanium or gallium arsenide by a factor of 2–10. Therefore, organic semiconductors have an intrinsic advantage due to the fact that they are highly efficient light absorbers. As a consequence, biointerface devices based on organics can be much thinner (based on films of tens to hundreds of nanometers) and lightweight and therefore minimally invasive when implanted. This capability of using less absorber material, combined with the relative mechanical flexibility and possible biocompatibility, makes organic semiconductors promising choices for light-activated interfaces. Despite these advantages, the use of organic light transducers for neurostimulation applications is a young and still emerging field, with examples of *in vivo* experiments being promising but still limited. In the following, we will consider first the mechanisms behind photostimulation, followed by discussion of some necessary context from *in vitro* examples and then, finally, *in vivo* validations themselves.

### 4.1 Photostimulation Mechanisms

There are three mechanisms by which organic light absorbers can transduce an optical signal into a bioelectronic one: (i) photothermal, (ii) photochemical, and (iii) photovoltaic. Photothermal can be further broken down into “simple” photothermal heating of a physiological medium or very rapid temperature changes which cause photothermocapacitive effects. Photovoltaic can likewise be separated into two different processes: photocapacitive, where light induces reversible formation of electrochemical double layers, and photofaradaic, where light drives redox reactions which may or may not be reversible. Photovoltaic mechanisms are the only ones which are analogous to “classic” electrical neurostimulation. Normal electrical neurostimulation relies on the injection of current from an electrode into a physiological medium, with the goal of altering the membrane potential of nearby excitable cells. To modify membrane potentials, the direction of current flow and the resultant electric field profiles must be carefully considered. These principles for current injection and flow apply also to the case of designing photovoltaic stimulation electrodes. As with traditional “wired” neurostimulation, the figure of merit is injected charge, or charge density. Charge density is the most useful metric for “macro” electrodes, of greater than 100 μm diameter. For microelectrodes often used in stimulation, thresholds for action potential usually scale not with density, but with total delivered charge.^80^ The injected charge (or charge density) is defined as the integral of current (or current density) over one phase of a stimulus waveform.^81^ Charge must be delivered rapidly, usually within 100–1000 μs, in order to stimulate voltage-gated sodium channels. This is because voltage-gated sodium channels, required for evoking action potential, have very rapid gating. The threshold values of charge for reproducible generation of action potentials (APs) will depend on the stimulation target. For example, *in vivo* stimulation of APs in peripheral nerves requires charge densities in the range 1–60 μC/cm^2^.^80−82^ For evoking AP in the retina, small microelectrodes are utilized to maximize spatial resolution of stimulation. Thresholds will vary depending on which types of excitable cells in the retina are targeted. Thresholds have been reported in the range from 0.05 mC/cm^2^ to as high as 1 mC/cm^2^, with thresholds generally being lower when larger stimulating electrodes are used.^83^

Photoexcited semiconductors dissipate absorbed energy via radiative or nonradiative processes. Nonradiative processes are either electrical work (as carriers are extracted into an external load, as in the case of a photovoltaic under normal operation) or heating. Semiconductors can be configured to suppress radiative and charge-generating effects and dissipate energy primarily as heat—which leads to a temperature increase of the semiconductor and its surrounding medium. There are two mechanisms by which photothermal heating can elicit an electrophysiological response: a slow process and a fast process. The slow process refers to the trivial effect of increased local temperature. Absorption of constant illumination lasting on the order of hundreds of milliseconds to seconds causes a temperature increase local to the site of illumination. Heat-sensitive ion channels, especially transient receptor vanilloid (TRPV) channels, can be activated by heat and lead to depolarization of excitable cells.^84^ There are a few examples in the literature of organic semiconductor/cell interfaces used to stimulate TRPV channels by photothermal heating. Colloidal nanocrystals synthesized from the pigment quinacridone were found to form close interfaces with cultured cells and be effective photothermal heating elements.^85^ Photoactivation of TRPV1 channels was measured with quinacridone/cell interfaces illuminated with green light pulses of 30 μJ energy dose.^85^ Photothermal heating in these interfaces was also found to increase currents flowing through potassium inward rectifier channels. Similar experiments have been performed with human embryonic kidney cells expressing TRPV1 channels which were photothermally activated with longer light pulses (tens to hundreds of milliseconds) using P3HT thin films as the light absorber.^86^ The effects of P3HT photothermal heating on single cells were evaluated as a function of irradiation time and intensity by Martino *et al.*(87) A completely different stimulation mechanism can arise from highly intense light pulses at a short time scale of 1 ms. Rapid local temperature increase of cell membranes causes their expansion, which results in a transient increase of cell membrane capacitance. A capacitance increase results in a depolarizing current. This effect was discovered when using direct heating of cells with intense infrared light pulses by Shapiro and Bezanilla *et al.*(88−90) The magnitude of the depolarizing current is proportional to the rate of temperature change and not the absolute change in temperature. For this reason, even with extremely intense laser pulses, when they are very short, they cause nonhazardous rises in temperature. A number of inorganic nano/microparticle light transducers have been used for photothermocapacitive stimulation interfaces; however, this effect has not been explicitly described for organic materials to date.^91^ Organic photothermal absorbers have been reported *in vivo* for photothermal cancer ablation;^92,93^ however, their deployment has been isolated to *in vitro* neurostimulation so far.

If the electrical potential across the semiconductor/electrolyte is high enough, photogenerated electrons and holes can be transferred to the electrolyte by respectively reducing or oxidizing molecules. Physiological electrolytes contain various molecules that can participate in redox reactions. Various organic molecules can be oxidized, such as sugars, or reduced, such as quinone molecules.^94^ Dioxygen, dissolved in water, is a potent electron acceptor. Moreover, water electrolysis is always a possibility, resulting in hydrogen or oxygen gas evolution. In the case of organic semiconductors, direct water splitting without transition metal cocatalysts has proved to be very inefficient and detectable only in trace amounts.^95^ In recent years, a number of studies have shown that oxygen reduction reactions are very common on organic semiconductor surfaces, and these can have important physiological effects.^96^ Single-electron reduction of O_2_ to superoxide^97,98^ or the two-electron reduction yielding hydrogen peroxide,^99−101^ H_2_O_2_, was demonstrated to proceed efficiently on a number of organic semiconductors. Both oxygen reduction reactions are thermodynamically favored over H_2_ evolution, with two-electron oxygen reduction to peroxide occurring at 0.7 V lower potential than H_2_ evolution. The dominance of oxygen reduction reactions was established in electrochemical,^102,103^ photoelectrochemical,^99,104^ and photochemical tests^98,101^ for polythiophenes, the biopigment eumelanin,^105^ and various crystalline organic molecular materials.^104^ These oxygen reduction products are known as reactive oxygen species (ROS) and have physiological effects ranging from toxicity at high concentration (>100 μM)^106^ to ion channel modulation^107^ and signaling effects (1 nM–0.01 mM range)^108^ at low concentrations. On-demand light-driven ROS generation by P3HT has been shown by Antognazza and co-workers to yield physiological effects both *in vitro*(109−111) and *in vivo*.^112^ The *in vivo* model chosen was the freshwater polyp, an eyeless animal. It was shown that P3HT colloidal nanoparticles were internalized by the polyp and photoirradiation resulted in behavioral changes as well as transcriptional changes in gene expression. Though not neuromodulation *per se*, this experiment showed that photogenerated ROS can be delivered by organic semiconductors and produce physiological changes.

These charge injection mechanisms are completely analogous to traditional neurostimulation; only the provision for light excitation “photo” is added. Currents flowing across an electrolyte will result in potential differences in the medium, which can depolarize cell membranes. Photoexcited charges in a semiconductor can travel to the semiconductor/electrolyte interface and cause charging of an electrolytic double layer. This phenomenon is regarded as photocapacitive charging. If the potential difference across this interface is sufficiently large, this charge can be transferred to a species in solution, resulting in a redox reaction. This electrical current originating from redox charge transfer is referred to as photofaradaic current. For neurostimulation applications, both capacitive and faradaic charge injection processes are acceptable, providing that the latter are fully reversible.^113^ In order for photocapacitive or photofaradaic currents to be injected by a semiconductor (organic or otherwise) into a physiological electrolyte, there must be a discrete cathode and anode component of the semiconductor/device in contact with the electrolyte. That is, there must be a path for the capacitive or faradaic current to flow through the solution in such a way as to generate potential differences that adjacent cells will experience. A number of photovoltaic configurations for photocapacitive^114^ and photofaradaic^115^ charge injection have been reported.

### 4.2 Photoactivated Interfaces *In Vivo*

Organic electronics for photo-driven neurostimulation applications is a relatively young field, with the first examples of *in vitro* work being reported within the past decade. Examples *in vivo* remain rare, though with the speed of developments at the *in vitro* level this is likely to change in the near future. Organic photointerfaces deployed to date *in vivo* can be divided into two application targets: the retina of the eye and subcutaneous implants for stimulation of peripheral nerves. Light-driven stimulation approaches have been explored the most in the context of artificial stimulation of the retina. Retinal prosthetic devices are aimed to restore partial visual sensitivity to patients afflicted with blindness conditions which are caused by degeneration of photosensitive cells but where the neuronal cells of the retina remain viable. Synthetic light-absorbing elements can be imagined to convert light into signals which stimulate the intact neurons of the retinal tissue and restore visual perception. Several retinal prosthetic devices are on the market and at various phases of clinical trials. There remains a substantial interest in making retinal stimulation approaches simple and more efficacious than the state of the art, and organic semiconductors have attracted interest to achieve this.

P3HT, by virtue of its widespread use as a photovoltaic polymer, ease of solution processability, and aforementioned high absorbance coefficient, is the most explored organic photoactive material for neurostimulation interfaces to date. Lanzani, Benfenati, Antognazza and colleagues published a comprehensive volume of work from 2009 to 2020, detailing the deployment of P3HT for neurostimulation in various *in vitro* settings and *in vivo* for retinal stimulation.^86,116−121^ This series of studies began with detailing the fabrication and measurement of organic photoelectrodes in contact with electrolyte. These photoelectrodes were characterized in the context of photovoltaic stimulation, with the hypothesis that photocapacitive currents would be injected into the physiological electrolyte. In 2013, Ghezzi *et al.* reported that films of P3HT (230 nm thickness) on conducting ITO substrates could elicit action potentials reliably in cultured neurons. In these experiments, hippocampal neurons are grown on P3HT substrates and then illuminated with 20 ms pulses with an intensity of 15 mW/mm^2^, corresponding to a light energy dose of 30 mJ/cm^2^. Action potentials are generated reproducibly with stimulation frequencies of 1–20 Hz, with the action potential probability falling from +90% for 1 Hz stimulation to around 70% by 20 Hz. Degenerated retinal tissues were then measured *ex vivo*, and action potentials were reliably triggered with light intensities at 4 mW/mm^2^. Follow-up work from 2015 showed that P3HT interfaces had a concurrence of three different effects: photocapacitive, photothermal, and photothermocapacitive. The photocapacitive effect and photothermocapacitive effect could both be implicated in the depolarization of cells and therefore stimulation of action potentials.

**Figure 5:**
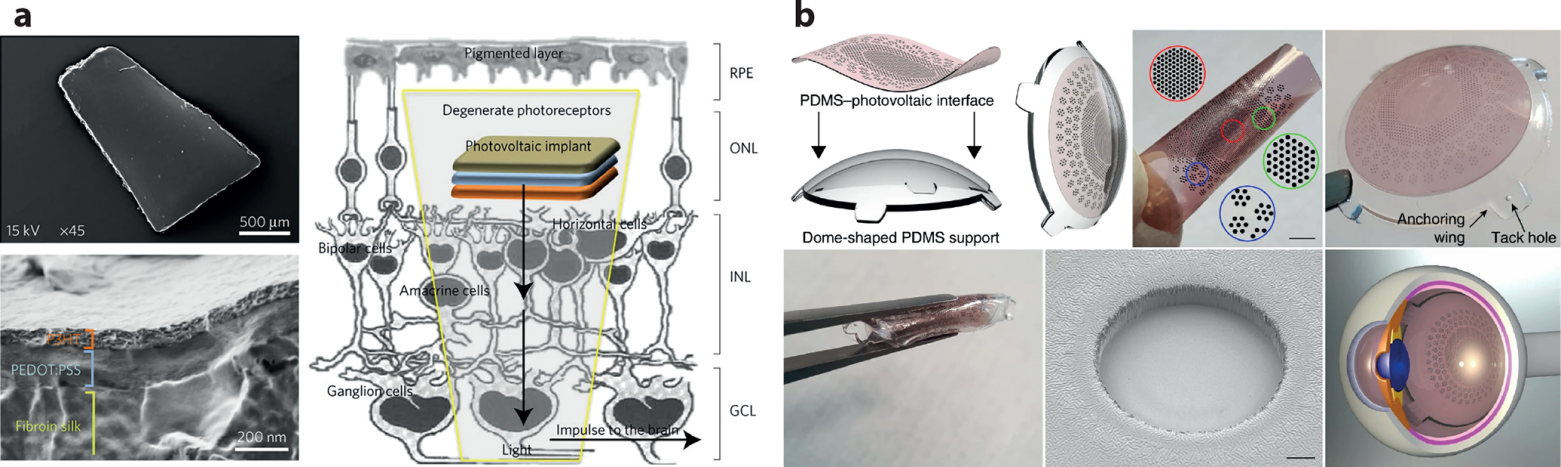
(a) P3HT-based retinal stimulation implantable devices which were shown to partially restore visual sensitivity to blind rats. Implantation was carried out for periods of 6–10 months. (b) P3HT-based photovoltaic pixels integrated into a foldable prosthetic implant for minimally invasive implantation into the eye. Part a reproduced with permission from ref (119). Copyright 2017 Springer Nature. Part b from ref (122). CC BY 4.0.

In 2017, the P3HT photoelectrode system was reported *in vivo*, with the possibility of stimulation of retinal tissues tested for the first time. The photostimulation implant consisted of a trilayer of silk fibroin serving as a biocompatible substrate, PEDOT:PSS as an underlying conducting layer, and P3HT as a charge-generating material and capping layer (Figure 5a). The devices were implanted subretinally in dystrophic rats. This animal model shows vision impairment in terms of both light sensitivity and spatial acuity and is an established model for degenerative blindness. The efficacy of stimulation by the device was validated using recording of visually evoked potentials (VEPs), measuring pupillary reflex, as well as behavior assessments. Implantation was tracked over 6 months, after which devices were explanted and characterized. The explanted devices were found to preserve a similar level of photoelectrical charging and upon microscopic inspection appeared to be fully intact and undegraded. In parallel, there was exploration of using colloidal P3HT nanoparticles (in the range of hundreds of nanometers in diameter) for neurostimulation. In 2020, it was reported that subretinally injected colloidal dispersions of P3HT could achieve similar *in vivo* effects as the planar devices published in 2017. The approach is attractive as such injection is surgically more facile. On one hand, the *in vivo* compatibility of P3HT appears promising, and there is evidence that neurostimulation can be induced by P3HT, at least at relatively high light intensities. On the other hand, there are still open questions related to the mechanisms at play: whether P3HT can stimulate via a photovoltaic mechanism or whether photochemical mechanisms or photothermal mechanisms are predominantly responsible for the observed electrophysiological effects *ex vivo* and *in vivo*. An important point of distinction is that the to-date-reported P3HT devices do not incorporate a structure of a primary electrode and return electrode as normal neurostimulation configurations do; therefore, there is not a clear current path for photovoltaic faradaic or capacitive currents to flow. Commercial availability, ease of processability, and indications for biocompatibility make P3HT and related polythiophenes an interesting choice for functional photostimulation interfaces. Issues that remain to be solved are engineering devices to deliver high currents/current densities and also material stability, which under photoirradiation conditions may not be sufficient for chronic stimulation. Recently, P3HT-based organic photovoltaic cells have been incorporated into a soft multielectrode array of stimulation electrodes of 130 μm diameter (Figure 5b).^122^ The entire array is foldable, enabling a minimally invasive implantation into the eye. This was only carried out on surgical phantoms as yet; however, the idea, combined with higher-performance organic photovoltaic pixels, holds high potential for future implantable stimulators.

**Figure 6:**
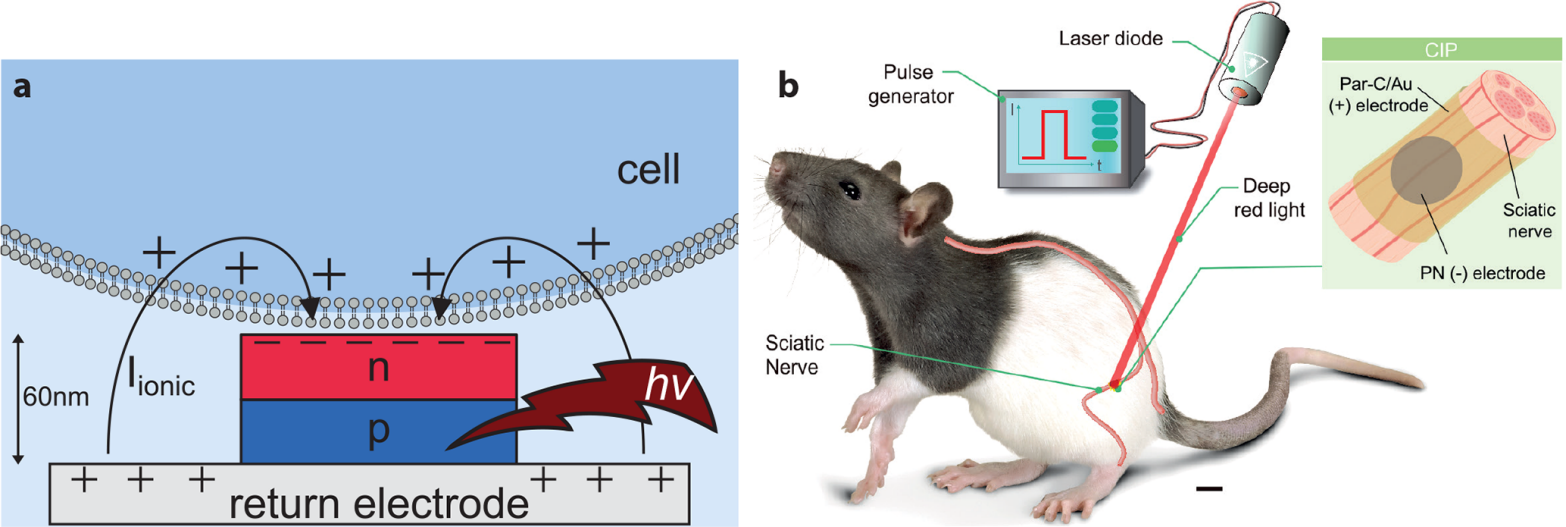
(a) Organic electrolytic photocapacitor (OEPC) capacitive coupling to adjacent cell membranes. The OEPC consists of a charge-generating p–n junction atop a return electrode. Ionic currents short-circuit the device over the electrolyte during charging/discharging. (b) *In vivo* OEPC implants for stimulation of the sciatic nerve prove that organic photovoltaic implants can function under chronic conditions and can be powered using tissue-penetrating red light. Part b reproduced with permission from ref (127). Copyright 2020 Silverå-Ejneby *et al.*

The OEPC was introduced in 2018 as a thin film photoelectrode device to mimic established bipolar electrode stimulators.^123^ The original OEPC features a bilayer donor–acceptor photovoltaic structure, fabricated via vacuum sublimation of H_2_Pc as the electron donor, and a perylene tetracarboxylic diimide derivative, PTCDI, as an electron acceptor. The system of materials was chosen as such evaporated bilayers are well-established in the literature and stand out for operational stability. The OEPC p–n donor–acceptor junction functions as the charge-generation layer as well as the primary stimulation electrode. The OEPC transduces impulses of light into ionic currents flowing in solution. The process proceeds as follows: A light impulse is absorbed by the p–n layer and electrons travel to the n-type material/electrolyte interface, while holes are injected into an underlying metallic electrode (Figure 6a). As a consequence, two electrolytic double layers are formed: one on the n-type/electrolyte interface and the other at the back contact/electrolyte interface. The former is cathodically polarized, while the latter is anodically polarized. Ionic current will flow in the surrounding solution while the two respective double layers are charging up. Ionic current can only flow in solution at time periods shorter than the charging time of the double layers and will necessarily be limited by the double layer with the smaller capacitance.^114^ Sustained ionic direct currents would only be possible if both cathode and anode components support faradaic reactions.^124,125^

The OEPC was tested for stimulation *in vitro* with single cells, primary neuronal cultures, and explanted retinal tissues.^114,123,126^ In these applications, the OEPC device was able to stimulate via a photovoltaic mechanism that was photocapacitive, using light intensities on the order of 1–8 mW/mm^2^ which generated 1–2 μC/cm^2^ over 1 ms of illumination. OEPCs were reported *in vivo* in 2020 for transcutaneous stimulation of peripheral nerves.^127^ Silverå-Ejneby *et al.* showed how OEPCs can be fabricated on parylene (3 μm thick) modified with thin, semitransparent gold (Figure 6b). These flexible OEPC stimulators were integrated into a zip-tie locking mechanism so as to be chronically implanted around the nerve. In this study, the sciatic nerve in rat served as a model to validate direct photoelectrical stimulation. The OEPC devices could be implanted at a depth of roughly 1 cm below the surface of the skin and actuated transcutaneously by using a 638 nm laser diode over the course of 100 days. Validation was performed by shining impulses of light at the implant (100–1000 μs pulse length) and measuring electromyography (EMG) of the leg and paw. EMG signals were accompanied by clear leg movements, evidencing direct photoelectrical stimulation of the sciatic nerve. This approach to ultrathin nerve stimulators is an application where the mechanical and optical properties of organic semiconductors can be highly competitive.

## 5 Flexible and Stretchable Stimulating Electrodes

The mechanics and form factor of neuromodulation devices are of general importance for all types of devices, ranging from conventional electrodes to photoactivated electrodes and drug delivery devices. When the mechanical aspects of various devices are described, the terms “soft”, “flexible”, and “stretchable” are often used, sometimes interchangeably. “Soft” refers to a material property, often the elastic modulus, while “flexibility”, the ability to bend, is the result of both softness and geometry. A stretchable material or device can be elongated, often in an elastic manner, while remaining functional. This typically requires tolerance to higher levels of strain within the materials than for flexible devices. To achieve a chronically stable neural interface, flexibility or stretchability is often required for surface electrodes interfacing peripheral nerves or the spinal cord.^128,129^ In the case of penetrating neural probes, they should not induce a severe tissue response or migrate within the tissue over time. It is known that a mechanical mismatch between the neural probe and the soft neural tissue (*E* < 10 kPa for brain) can cause both issues, as natural bodily movements, respiration, and vascular pulsatility induce movements within the tissue.^130−132^ The compliance of a rectangular probe is characterized by its axial stiffness *k*_A_ = *Ewt*/*l* and bending stiffness *k*_B_ = *Ewt*^3^/*l* (elastic modulus *E*, width *w*, thickness *t*, length *l*). The advantage of thin polymer probes can be understood on the basis of the bending stiffness, as thickness is the most important property to achieve low bending stiffness, i.e., flexibility. For axial stiffness (elongation), thickness is less important, and a low elastic modulus is necessary to accommodate tissue motions.^133^ The tunability of the mechanical properties of conducting polymers makes them attractive for flexible and stretchable neural interfaces. Various formulations of PEDOT:PSS have gained the most attention, likely due to their superior chemical stability. Pristine dry PEDOT:PSS is rather hard and brittle, with an elastic modulus in the gigapascal range and a fracture strain around 2–6%.^134^ The swelling behavior of PEDOT:PSS in water depends strongly on the processing conditions and additives. PEDOT:PSS with the addition of 5% ethylene glycol has been reported to have an elastic modulus in the 100 MPa range in the swollen state.^135^ The addition of GOPS (3-glycidoxypropyltrimethoxysilane), a common stabilization additive, increases the wet elastic modulus to ∼300 MPa.^135^ A variety of additives can be used to soften PEDOT:PSS films and improve their stretchability, for example, polyethylene glycol.^136^ To reach really soft mechanical properties, conductive hydrogel formulations are employed.^137,138^

**Figure 7:**
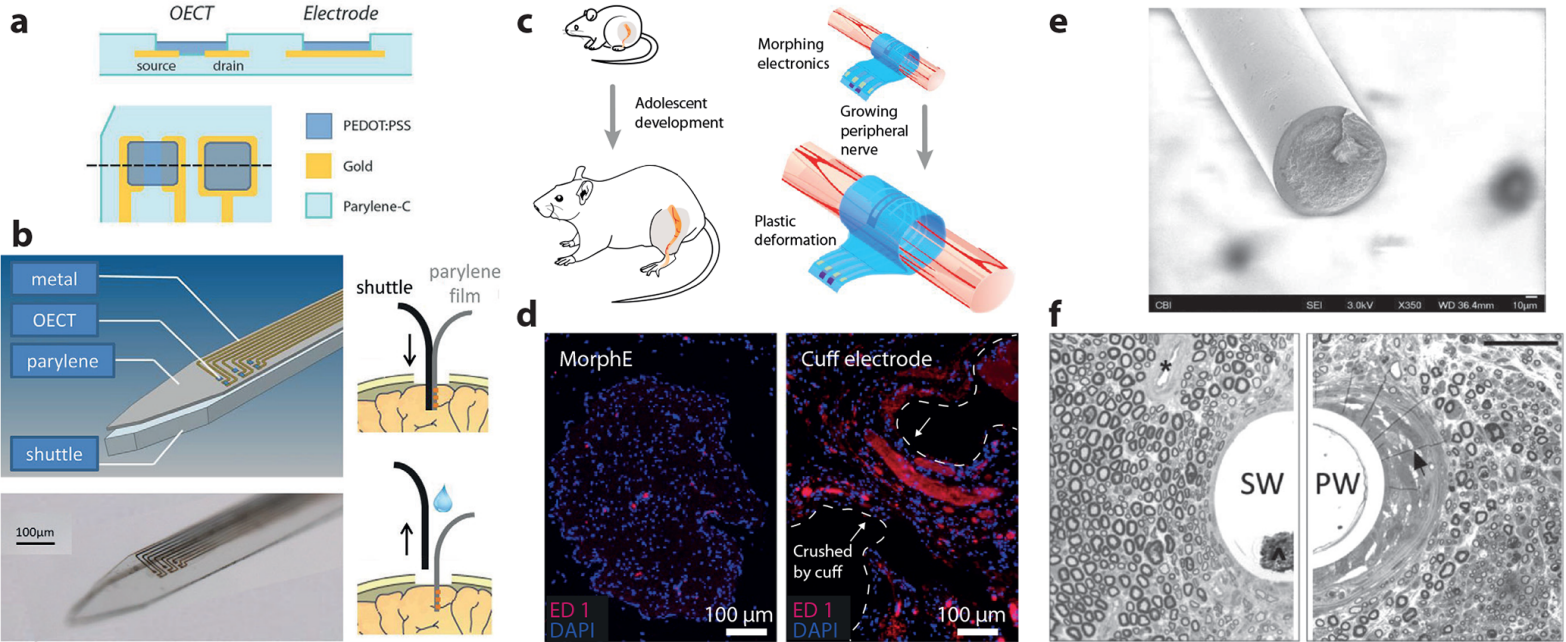
Flexible and stretchable conducting polymer based devices for neuromodulation. (a) PEDOT:PSS electrodes and OECTs were integrated into the bending plane of a 4 μm thick parylene C device. (b) The ultraflexible probe was inserted with a stiff shuttle.^140^ (c) The viscoplastic PEDOT:PSS electrode could expand along with the growing tissue. (d) The viscoplastic electrode (MorphE) induced little inflammation (inflammatory biomarker ED1) in comparison to a conventional cuff electrode. (e) Soft and stretchable microwire based on a silicone/PEDOT–PEG/CNTs composite. (f) Histology of chronically implanted nerves with the soft microwire (SW, left) and polyimide wire (PW, right). The soft wire induced less scar tissue around the wire. Parts a and b reproduced with permission from ref (140). Copyright 2015 John Wiley and Sons. Parts c and d reproduced with permission from ref (146). Copyright 2020 Springer Nature. Parts e and f reproduced with permission from ref (148). Copyright 2019 John Wiley and Sons.

Parylene, polyimide, and SU-8 have been the most popular substrate and insulation materials for flexible neural interfaces. The total device thickness plays an important role here, as the bending stiffness is 1000 times higher for a 20 μm thick device in comparison to a ultraflexible 2 μm^139^ thick device. Williamson *et al.* developed a 4 μm thick parylene probe with gold conductors and PEDOT:PSS electrodes and electrochemical transistors (Figure 7a,b).^140^ Due to its ultraflexibility, the probe had to be attached to a rigid shuttle during insertion, after which it was delaminated and the shuttle was removed. The device could stimulate local populations of neurons and record activity with the transistor, while limiting the tissue response due to its outstanding flexibility. Boehler *et al.* developed an alternative approach for flexible neural probes as they included Dex-loaded PEDOT electrodes onto the probes.^54^ The Dex could be actively released by electrical addressing of the drug-loaded PEDOT electrodes, which showed a positive effect during 12 weeks of implantation with respect to the proximity of neurons to the electrodes. Flexibility also allows for the development of nonplanar device geometries for various applications. Ferrari *et al.* combined ink jet printing of PEDOT:PSS with a heat-shrinkable polymer substrate to form cuff electrodes for nerve regeneration applications.^141^ The device was able to stimulate regenerated motor axons to induce a muscular response 3 months after implantation. In another approach, Tian *et al.* used the flexibility of their parylene C–PEDOT:PSS microelectrodes to create a flexible tubular electrode with drug delivery capability.^142^

A recent trend is to go beyond the limitations of flexibility by developing soft and stretchable conducting polymer based stimulation devices. To achieve stretchability, all device layers must be stretchable or arranged geometrically so that they are isolated from strain. A special class of highly elastic polymers, elastomers, are therefore used as substrates and encapsulation for such devices. There exist a variety of different elastomers, although only a small portion of those are suitable for biomedical use and implantation.^143^ Stretchable conducting polymers can be achieved by tailoring the material structure in combination with swelling in water^137^ or by forming conducting polymer–elastomer composites.^144^ However, even modified conducting polymers typically have inferior robustness and stretchability in comparison to many elastomers. It is therefore of the utmost importance to create good adhesion between the substrate/encapsulation and the conducting polymer layer to achieve robust and stretchable devices. As many elastomers are hydrophobic and have low surface energy, activation by, e.g., oxygen plasma is often used to improve the adhesion between the layers.^145^ Qi *et al.* developed stretchable highly conductive (∼800 S/cm) PPy–toluenesulfonic acid conductors by the use of prestrained PDMS substrates.^500^ The anchoring of the PPy conductors to the PDMS was facilitated by the fabrication of PPy nanowires as an adhesion layer, which greatly improved the adhesion to the PDMS substrate. Stretchable multielectrode arrays were used for acute recording of electrocorticographic signals and stimulation of the sciatic nerve in rats. To achieve even softer stretchable electrode arrays, Liu *et al.* developed PEDOT-based hydrogel conductors (∼50 S/cm) by dissolving an ionic liquid additive out of the conductors after the film formation. Soft (∼30 kPa) cuff electrodes were developed, based on the conductive hydrogel and a fluorinated substrate material, and chronically implanted around the sciatic nerve in mice. The soft cuff electrodes showed less tissue response and lower stimulation threshold than the commercial reference cuff electrode. By instead using glycerol as an additive to PEDOT:PSS, Liu *et al.* developed viscoplastic hydrogel electrodes (∼1 S/cm).^146^ Together with a viscoplastic polymer, electrodes that could expand along with growing tissue were achieved and implanted around the sciatic nerve of growing rats (Figure 7c,d). Penetrating probes are necessary to achieve higher specificity in peripheral nerve stimulation, but such probes typically induce a severe tissue response.^147^ Zheng *et al.* addressed this issue by developing stretchable microwire electrodes based on a silicone/PEDOT–PEG/CNTs (<5 S/cm) composite with an elastic modulus below 1 MPa (Figure 7e).^148^ The implant could evoke force and compound muscle action potentials in the tibial nerve of rats and showed less scar tissue encapsulation after 1 month of implantation in comparison to a polyimide wire (Figure 7f). The above examples demonstrate the benefits of soft and stretchable conducting polymer electrodes for neuromodulation; however, a limiting factor is the relatively low conductivities of these materials. It might therefore be beneficial to combine stretchable conducting polymers with high-performance stretchable inorganic conductors^129,149^ for the next generation of soft and stretchable devices for neural modulation.

## 6 Biohybrid Interfaces

Biohybrid neural interface devices combine principles of tissue engineering with bioelectronic technologies,^150−152^ aiming to minimize or even eliminate the foreign body response and to improve the communication and signal transduction between the biotic and abiotic interfaces. Tissue regeneration principles can be used to restore the damaged tissue but also to form a more natural connection between the device and the target tissue. For example, the use of autologous iPSCs, induced pluripotent stem cells, that are harvested from the patient will in principle suppress any immune response at the implantation site. However, studies have shown that iPSCs can have low viability after transplantation and even form tumors.^153^ On the other hand, neural stem cells (NSCs) have shown better viability and the ability to differentiate into both neurons and glia cells.^154^ In addition, NSCs secrete neurotrophic factors that promote the axonal regeneration in the host neurons while at the same time reducing glial formation and enhancing healing.^155^ Although the two fields have made significant progress independently over the past decades, biohybrid neural interface devices are at a very early stage of development with only a few examples in the literature for both the central and peripheral nervous systems.

### 6.1 Biohybrid Interfaces in the CNS

**Figure 8:**
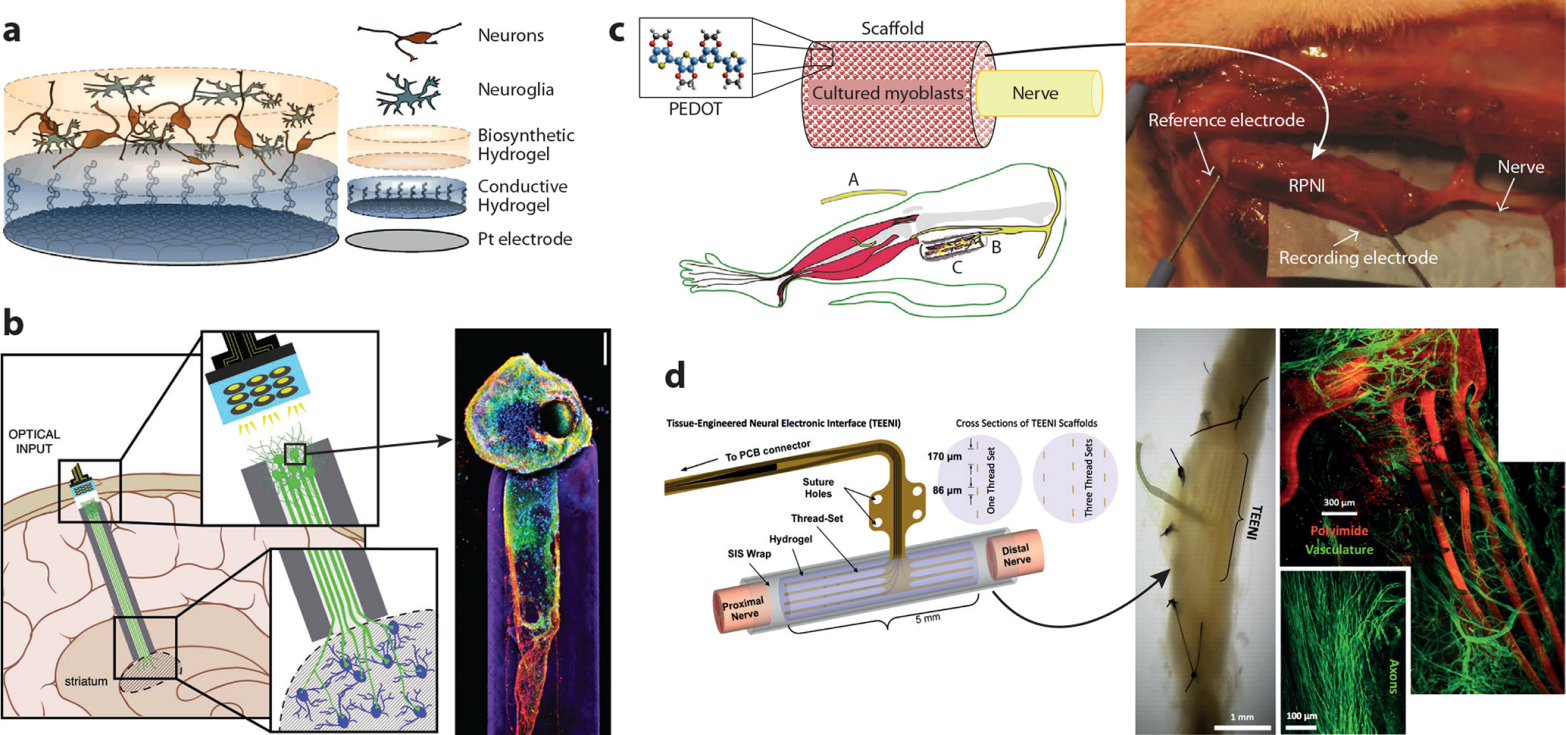
Examples of biohybrid neural interface devices. (a) Cell-seeded electrode consisting of Pt, conducting hydrogel, and hydrogel for culture of neuroprogenitor and glia cells. (b) (left) Living electrode concept where a neuronal axonal electrode is cultured *in vitro* within a columnar hydrogel with the potential to be injected in the brain and interfaced with a neuromodulation device. (right) Confocal reconstruction of a unidirectional, cerebral cortical neuronal living electrode at 11 days of culture *in vitro*, immunolabeled for axons (β-tubulin-III; red) and synapses (synapsin; green) with a nuclear counterstain (Hoechst; blue). The surrounding hydrogel microcolumn is shown in purple. (c) (left) Regenerative peripheral nerve interface (RPNI) that is based on a scaffold of acellular muscle coated with PEDOT that contains myoblasts and is wrapped around the end of the peripheral nerve. A section of the distal common peroneal nerve is removed (A), and the residual nerve (B) is implanted into the RPNI (C) for a minimum of 2 months. (right) *In situ* image of RPNI 4 months after implantation. (d) (right) Tissue engineered electronic nerve interface (TEENI): 16-channel device attached to the ends of a transected nerve. Insets show 1 mm diameter cross-sectional views of the construct with a single thread set (4) and a multiple thread set (3–4–3) arrangement. (left) Histological analysis of a TEENI device after a 6 week implantation. (left) Optical microscope image of an explanted nerve that regenerated through a TEENI hybrid scaffold with the microfabricated device visible inside the nerve. (top right) Light-sheet microscope image of a TEENI device (red) and the vasculature (green) inside a regenerated nerve. (center bottom) Image of regenerated axons within a TEENI device. Part a from ref (180). CC BY 3.0. Part b reproduced with permission from ref (181). Copyright 2018 John Wiley and Sons. Part c from ref (163). CC BY 4.0. Part d reproduced with permission from ref (182). Copyright 2019 IEEE.

Green *et al.* were the first to demonstrate the concept of a biohybrid electrode based on organic electronic materials^156,157^ (Figure 8a). The living electrode construct, as they named it, was a Pt electrode with a conducting hydrogel that supported neural progenitor and glia cell growth.^157^ The study focused on evaluating the electrode performance and cell viability *in vitro*. A PVA-based hydrogel was initially cross-linked on top of the Pt electrode, and then PEDOT was electropolymerized through the hydrogel in order to induce electronic conductivity. Then a macromer solution that contained neuroprogenitor or glia cells was deposited on top of the conducting hydrogel and cross-linked with UV in order to encapsulate the cells. Hydrogels are widely used materials for tissue engineering as they can mimic the mechanical properties of the *in vivo* environment and they can support cell growth with diffusion of nutrients and other signaling molecules. The electrode had initially a relatively high modulus of 140 kPa, but over time it became softer due to swelling, reaching 1.5 kPa after 21 days in an aqueous environment. In comparison with a neat Pt electrode, the living electrode construct had lower impedance (1–1000 Hz), higher charge storage capacity, and higher charge injection limit. Glia cells showed high viability, reaching 80% in the course of 7 days, while the viability of the neural progenitor cells was low. The neuroprogenitor cells were encapsulated immediately after harvest, and the authors speculate that the presence of cell debris might have negatively impacted the cells. The authors also evaluated the extracellular matrix production in an additional study and showed that both laminin and collagen were produced, with laminin distributed throughout the hydrogel while collagen was present only at the surface of cells. The cells, although they were distributed throughout the hydrogel, mostly formed aggregates. In this work the biohybrid electrode was only evaluated *in vitro*, and while the electrical performance of the electrode was promising, the cell growth within the hydrogel scaffold requires further optimization. Furthermore, the dimensions of the electrode should be miniaturized for *in vivo* testing.

The first *in vivo* evaluation of a cell-seeded probe was presented by Purcell *et al.*(155) The probe was based on SU-8 encapsulated in parylene C and had an opening along its length where NSCs were seeded in an alginate hydrogel. The study focused only on the cell viability, and the probe had no active sites. The tissue response after implantation in the cortex of rats was evaluated at four time points over 3 months. Up to 1 week post implantation, higher neural density was observed around the cell-seeded probe in comparison with controls, but after 6 weeks increased neuronal loss and glial encapsulation were observed. The initial positive effect could be a result of the neurotrophic and neuroprotective factors that are released by the NSCs,^158^ while the later negative effect could be due to reduced viability of the NSCs and/or a delayed immune response from the host tissue. In another work, NPCs were immobilized on laminin-coated Si neural probes.^159^ The cells were cultured *in vitro* for 14 days, showing growth and differentiation along the probe, and then the probe was implanted in murine cortex. At 1 and 7 days post implantation viable NSCs were detected on the probe and in its proximity. Furthermore, the authors observed a reduced glial response that could be the effect of secreted neurotrophic factors from the NSCs. Cell-seeded probes are still at a very early stage of development. While these examples show promise in terms of host tissue response, a more in-depth investigation is still required in order to demonstrate how the seeded cells and the host tissue integrate and communicate over time.

Taking the idea of cell-seeded probes a step further is the concept of the living electrode introduced by Cullen *et al.*(160) The idea is to form an axon-based electrode consisting of a neuronal tissue engineered construct that can be injected into the brain and act as a transducer between the host tissue and an external electrical or optical neuromodulation device that will lie on the surface of the brain (Figure 8b). Depending on the nature of the engineered neurons, the living electrode can have excitatory, inhibitory, or modulatory effect as it will be determined by the type of synapses that will be formed with the host tissue. The motivation behind this approach is to enhance neuromodulation through a biological interface that can have high specificity, high synaptic density, and long-term integration. So far living electrodes have been demonstrated *in vitro* based on columnar hydrogels that support neural growth and longitudinal axonal outgrowth of glutamatergic, dopaminergic, and GABAergic neuron subtypes.^161,162^ On the basis of the cell culture conditions and hydrogel composition, the length of the axons can vary between submillimeter and centimeter and, therefore, can target different areas in the brain after injection. In a preliminary *in vivo* study living electrodes with GFP-modified cortical neurons were microinjected between the cerebral cortex and the thalamus in rats.^162^ At 7 and 28 days post implantation the implanted neurons survived, maintaining the engineered architecture, while there were indications of formed synapses with host neurons based on the presence of the presynaptic protein synapsin in the proximity of the transplanted neurons.

### 6.2 Biohybrid Interfaces in the PNS

Biohybrid devices for interfacing with the peripheral nervous system also exist. Similarly to the CNS devices, the goal is to suppress the host response, in this case to minimize neuroma formation and axonal damage and to enhance device integration. Advanced prosthetics for example require high fidelity control with multiple channels of independent motor control and sensory feedback that can be achieved via a high-density cell connection.

In a proof-of-concept study Urbanchek *et al.* presented the regenerative peripheral nerve interface (RPNI), a device that is composed of a scaffold that supports differentiation of myoblasts and regeneration of nerves as a new strategy for connecting divided peripheral nerves with artificial limbs^163^ (Figure 8c). The scaffold consisted of an acellular muscle with chemically polymerized PEDOT. The distal end of a divided peroneal nerve was inserted in the cylindrical scaffold where myoblasts were cultured. The RPNI was evaluated on average 93 days after implantation. EMG activity was recorded from the RPNI, and the myoblasts within the construct developed into mature muscle that was reinnervated and revascularized without any indication of neuroma formation. Furthermore, neuromuscular junctions were detected, indicating formation of synapses between the regenerated axon and the myoblast derived muscle fibers. The PEDOT electrode surrounding the cultured cells opens the possibility for parallel stimulation/recording, but that was not explored in this initial study.

Another hybrid approach for the PNS is the tissue engineered electronic nerve interface (TEENI) that aims to integrate soft and flexible electrode arrays into a hydrogel matrix that can act as scaffold for regeneration of nerves (Figure 8d).^152^ In this case the scaffold does not include any cells, but the idea is that host cells will grow within the scaffold and make an intimate connection with the electrode array. It was first presented by Desai *et al.*(164) with an electrode array based on flexible polyimide (PI) electrodes integrated within a pro-regenerative scaffold that was then wrapped in decellularized small intestinal submucosa. In a following work the TEENI was implanted in a damaged rat sciatic nerve with the distal and proximal nerves placed at the two ends of the device. After only 4 days of implantation, the authors recorded single unit activity at the distal electrodes and were able to record electrophysiological activity over 6 weeks.^165^ The TEENI was well integrated into the tissue with vascularization and axons throughout the scaffold. A more detailed immunohistochemical analysis was performed in another work and showed the presence of regenerated axons and Schwann cells but also a foreign body response related to the presence of the PI electrode threads.^166^

## 7 Piezoelectric Stimulation

**Figure 9:**
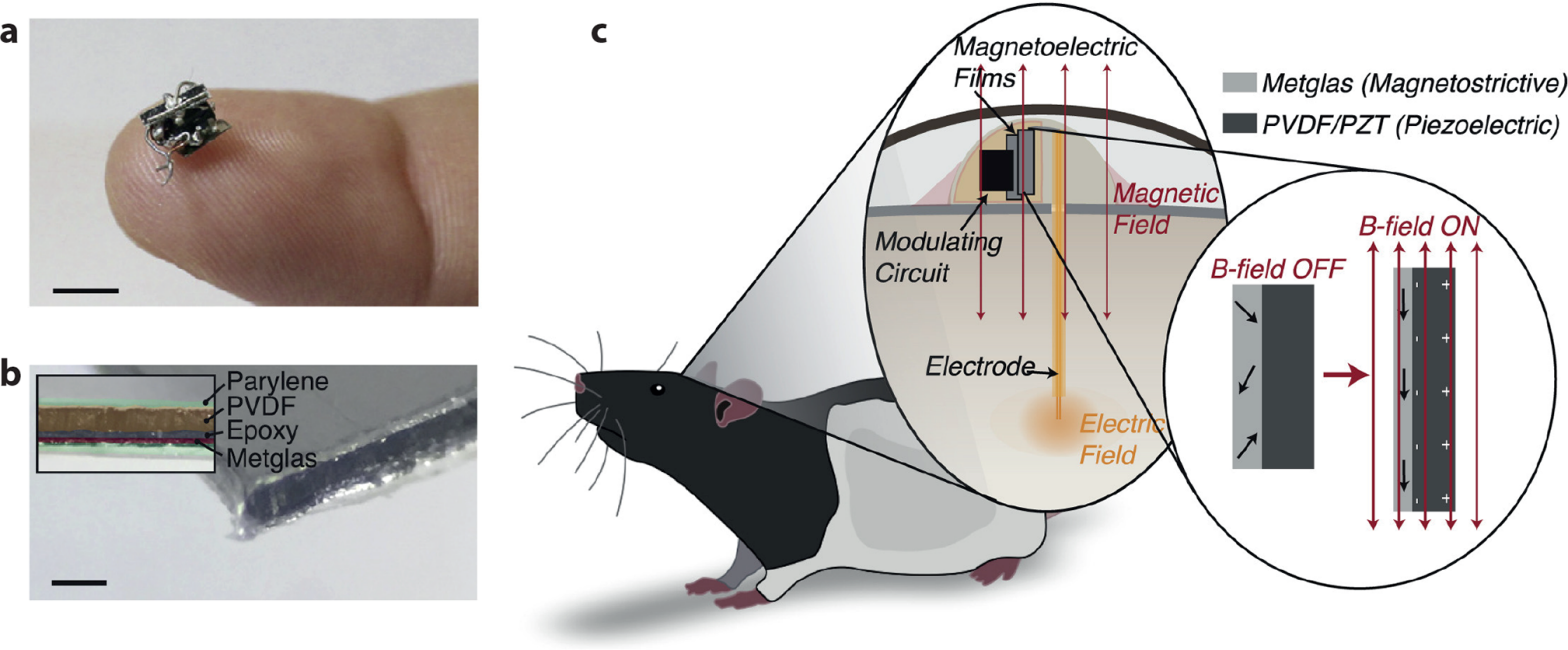
Piezoelectric thin films coupled with magnetostrictive materials allow for transduction of oscillating magnetic fields to resonant mechanical vibrations which in turn are converted by piezoelectric PVDF into alternating AC currents capable of direct neurostimulation. By using two different resonant frequencies and a diode bridge circuit, biphasic stimulation can be accomplished. Reproduced with permission from ref (168). Copyright 2020 Elsevier.

Piezoelectric materials generate a voltage upon mechanical deformation, resulting in current flow. It is possible therefore to extract power from a piezoelectric material. Periodic mechanical vibration can thus be transduced to alternating electrical currents, which can then be applied for neurostimulation. A number of organic polymeric materials are well-established piezoelectrics. The most prominent is PVDF (polyvinylidene fluoride),^167^ which is used in numerous commercial electronic products and has likewise found its way into *in vivo* stimulation devices. Singer and co-workers recently reported the concept of magnetoelectric transduction for neurostimulation (Figure 9).^168^ The process relies on PVDF for generating an AC current. PVDF is coupled with a magnetic strip which in turn vibrates with a resonant frequency excited by an external magnetic field. Therefore, a magnetic field generates a mechanical vibration, which is transduced by PVDF into AC current. Due to the excellent tissue-penetrating properties of magnetic fields, this kind of wireless stimulator has the potential for interfacing to deep targets, for example in the brain. The device consists of a thin film of PVDF which is laminated with Metglas SA1, a magnetostrictive alloy, and is encapsulated in parylene C, giving a minimal mechanical form factor. An alternative pathway is to couple acoustical waves directly to activate a piezoelectric material, which is the concept behind actuating implanted piezoelectric stimulators with focused ultrasound.^169,170^ The focused ultrasound/piezoelectric approach has gained increasing attention recently; however, inorganic piezoelectric crystals are used. The thin film form factors enabled by polymeric piezoelectrics such as PVDF may enable more minimalistic and biocompatible stimulators in the future.

## 8 Conclusions and Outlook

Using organic electronic materials for *in vivo* bioelectronic interfaces offers a high degree of versatility due to the synthetic tunability of material properties. There is an increasing amount of experimental indications that organic electronic materials have good potential biocompatibility. The mechanical softness, relative to traditional ceramic, inorganic materials, can reduce the foreign body response upon implantation. On the other hand, bioorthogonality is desired. The chemical similarity between organic conductors and biology may not always be ideal for the living system. To date, the long-term effects of implanting organic electronic materials remain poorly understood. The most important outlook for this field of research is to establish the suitability of organic electronic materials for chronic applications. PEDOT-based materials have shown very promising performance for *in vivo* recording, for example in the CNS. Likewise, the use of organic electrodes like PEDOT has seen increased attention for stimulation applications in recent years,^171^ with efforts focusing on understanding stimulation efficiency,^172^ material stability,^173−175^ and improving long-term device performance.^176^ However, due to the lack of large patient studies utilizing such organic material systems, chronic safety remains an open question.

While organic materials have advantages as active bioelectronic interface components, their mechanical properties, and the role of organic substrates and encapsulants, are equally critical for successful neural interfaces. A wide range of elastic moduli can be achieved for conducting polymers, from gigapascal in the pristine state to kilopascal in conductive hydrogel formulations. This enables matching of the mechanical properties of interfaced tissues, which is of especial importance when interfacing deforming tissues in the PNS. The chemical structure of organic electronic materials also enables innovative approaches for the deployment of stimulation electrodes. Conducting polymers can be formulated into injectable dispersions that solidify *in situ*. The molecular nature of the conductors can also enable specific interactions with, and the bridging of, biological structures. Furthermore, gentle polymerization approaches may allow for *in situ* formation of integrated conductive structures. Altogether, organic electronics promise unparalleled integration with tissue and thereby specific neuromodulation and recording.

The neurostimulation field is increasingly pushing toward wireless solutions to enable less invasive stimulation *in vivo*. In line with this, the possibility of exploiting efficient light absorption by organic semiconductors has created the nascent field of organic optobioelectronic interfaces and stimulators. The amount of work *in vivo* remains very limited; however, the increasing diversity of organic stimulation devices tested under *in vitro* conditions indicates that this field is set to grow. A major question which must be confronted by the field is, once again, stability. Researchers often publish validations of successful performance but fail to carry out and report device stability.

Organic electronic materials have great potential for controlled delivery of drugs and biomolecules, as their structure allows for the incorporation and transport of molecular entities. As chemical stimulation is very versatile and typically operates in the low-frequency regime, it constitutes an attractive complement to high-frequency electrical stimulation. Organic electronic ion pumps have shown considerable promise for *in vivo* deployment, yet important questions must be answered regarding chronic stability and compatibility. Solutions for the replenishment of reservoirs must also be found, or the ion pump technology must focus on applications that require relatively low doses and/or only short-term application.

Overall, the field of *in vivo* organic bioelectronics is just beginning to gain larger interest and acceptance, as many materials and device concepts are validated at the *in vitro* stage and mature to the level of *in vivo* applications. Indeed, medical devices incorporating organic electronic materials (coatings) have just recently been awarded FDA and CE approval, e.g., Acutus Medical’s AcQMap system^177^ incorporating Heraeus’s Amplicoat PEDOT formulation.^178^ Heraeus (one of the largest producers of PEDOT and related derivatives) has confirmed^179^ that they are currently working on commercialization of several additional applications for Amplicoat—including neuromodulation—but only the AcQMap system has reached the level of FDA/CE approval. An important caveat to the push for medical approval and *in vivo* or even in-human testing is that scientists working in this field should consider thorough characterization of materials and devices *ex vivo*, especially with regard to stability and reliability. This is important from the point of view of ethical use of laboratory animals, where scientists should optimize their approaches as much as possible before planning *in vivo* experiments.

Altogether, organic electronics materials and systems already present promising capabilities for neuromodulation technology. As preclinical demonstrators are translated into clinical devices, we feel certain that patients in the not-too-distant future will benefit from such organic bioelectronic technologies. This being said, the *in vivo* chronic test of time is the next frontier for organic bioelectronics to prove itself for wide-scale acceptance in neuromodulation protocols.
